# The association of upper airway anatomy with cognitive test performance: the Multi-Ethnic Study of Atherosclerosis

**DOI:** 10.1186/s12883-023-03443-9

**Published:** 2023-10-31

**Authors:** Robin M. Nance, Alison E. Fohner, Robyn L. McClelland, Susan Redline, R. Nick Bryan, Annette Fitzpatrick, Mohamad Habes, WT Longstreth, Jr., Richard J. Schwab, Andrew S. Wiemken, Susan R. Heckbert

**Affiliations:** 1 University of Washington, 325 9th Ave, Box 359931, Seattle, 98104 USA; 2 Department of Epidemiology & Cardiovascular Health Research Unit, University of Washington, Seattle, USA; 3 University of Washington, Seattle, USA; 4 Brigham and Women’s Hospital, Harvard Medical School, Boston, USA; 5 Department of Radiology, University of Pennsylvania, Philadelphia, USA; 6 Neuroimage Analytics Laboratory and Biggs Institute Neuroimaging Core, Glenn Biggs Institute for Neurodegenerative Disorders, University of Texas Health Science Center at San Antonio, San Antonio, TX USA; 7 Departments of Neurology and Epidemiology, University of Washington, Seattle, USA; 8 Department of Medicine, University of Pennsylvania, Philadelphia, USA

**Keywords:** Upper airway anatomy, Cognitive function, Machine learning

## Abstract

**Background:**

Numerous upper airway anatomy characteristics are risk factors for sleep apnea, which affects 26% of older Americans, and more severe sleep apnea is associated with cognitive impairment. This study explores the pathophysiology and links between upper airway anatomy, sleep, and cognition.

**Methods:**

Participants in the Multi-Ethnic Study of Atherosclerosis underwent an upper airway MRI, polysomnography to assess sleep measures including the apnea-hypopnea index (AHI) and completed the Cognitive Abilities Screening Instrument (CASI). Two model selection techniques selected from among 67 upper airway measures those that are most strongly associated with CASI score. The associations of selected upper airway measures with AHI, AHI with CASI score, and selected upper airway anatomy measures with CASI score, both alone and after adjustment for AHI, were assessed using linear regression.

**Results:**

Soft palate volume, maxillary divergence, and upper facial height were significantly positively associated with higher CASI score, indicating better cognition. The coefficients were small, with a 1 standard deviation (SD) increase in these variables being associated with a 0.83, 0.75, and 0.70 point higher CASI score, respectively. Additional adjustment for AHI very slightly attenuated these associations. Larger soft palate volume was significantly associated with higher AHI (15% higher AHI (95% CI 2%,28%) per SD). Higher AHI was marginally associated with higher CASI score (0.43 (95% CI 0.01,0.85) per AHI doubling).

**Conclusions:**

Three upper airway measures were weakly but significantly associated with higher global cognitive test performance. Sleep apnea did not appear to be the mechanism through which these upper airway and cognition associations were acting. Further research on the selected upper airway measures is recommended.

**Supplementary Information:**

The online version contains supplementary material available at 10.1186/s12883-023-03443-9.

## Background

About 25% of adults over the age of 50 in the US have cognitive impairment [1], and due to the large size of the affected population, understanding risk factors for this impairment is critical. An estimated 26% of Americans over age 30 have sleep apnea [2], and prior research suggests that more severe sleep apnea is associated with cognitive impairment [3]. Understanding the pathophysiology and links between upper airway anatomy, sleep measures, and cognition may help facilitate treatment and prevention of the adverse outcome of poorer cognitive function. Treatments can be tailored based on the associations discovered, such as oral appliances if craniofacial (bony) morphology is an important exposure and uvulopalatopharyngoplasty surgery if soft palate volume is a critical exposure [4].

Upper airway anatomy, including craniofacial and pharyngeal soft tissue structures, may be associated with cognitive function given the role of upper airway anatomy in sleep apnea risk, as sleep apnea is associated with impaired cognition [5–9]. The specific association between upper airway anatomy and cognitive function is unknown [5–8]. Numerous upper airway anatomy characteristics have been reported as risk factors for sleep apnea, and variation in these characteristics may help identify people with sleep apnea who have more severe downstream outcomes [5, 6]. Studies included in two meta-analyses did not all find the same upper airway measures to be associated with sleep apnea, but many of them found associations with greater soft palate and tongue area [5, 6].

Cognitive function is postulated to be negatively impacted by sleep apnea, and many studies find that sleep apnea is associated with poorer cognitive function in certain domains but not others [9]. One study did not find an association between the apnea-hypopnea index and eight different measures of cognitive function [10]. A large meta-analysis reported an increased risk of progressing to cognitive impairment and poorer executive function among people with sleep disordered breathing, but found no difference in memory or global cognition domains [9]. Accordingly, evidence is conflicting on whether and which aspects of cognitive function are impacted by sleep apnea. Due to these previously reported associations, we hypothesized that lower tongue volume and lower soft palate volume would be associated with better cognition.

Using magnetic resonance imaging (MRI)-based measurements of upper airway anatomy in the setting of a large, multi-ethnic, deeply phenotyped cohort study, this analysis assessed the association of upper airway measures with performance on four cognitive function tests that measure different domains of cognitive function. There are numerous measures of upper airway anatomy, and it is unknown which, if any, are associated with cognitive test performance. Therefore, we used model selection techniques to select from among over 60 upper airway measures those that are most strongly associated with the four cognitive function test scores. Exploring the association of selected upper airway anatomy measures with cognitive test performance, both alone and after adjustment for sleep study measures, may help elucidate whether and reasons why upper airway anatomy is a potential risk factor for the adverse outcome of cognitive impairment.

## Methods

### Study design/setting/subjects

The Multi-Ethnic Study of Atherosclerosis (MESA) enrolled a racially and ethnically diverse cohort of 6814 men and women from six areas of the United States, including Black, Chinese-American, Hispanic and White participants [11]. At baseline in 2000–2002, all participants were between 45 and 84 years old and had no history of cardiovascular disease.

Extensive data collection was done at baseline and included self-reported preferred language, education, and a blood draw with samples stored for later use and genotyping. Follow-up study visits have occurred every 2–6 years, including exam 5 in 2010–2012 and exam 6 in 2016–2018. In conjunction with exam 5 (2010–2013), 2261 MESA participants underwent a sleep assessment including in-home polysomnography, actigraphy, and a sleep questionnaire using the Women’s Health Initiative Insomnia Rating Index and Epworth Sleepiness Scale [12]. Measures of sleep extracted from the polysomnography results included sleep architecture, time in each sleep stage, and measures of nocturnal hypoxemia, most importantly the apnea-hypopnea index (AHI). For this analysis, AHI was defined as the number per hour of apneas or hypopneas associated with at least a 4% oxygen desaturation plus the number per hour of arousals.

At exam 6, a median of 6 years after the sleep study, a subset of participants included in the MESA Atrial Fibrillation ancillary study [13] completed four cognitive function tests: the Cognitive Abilities Screening Instrument (CASI, version 2), the Digit Symbol Coding Test, the Digit Span (DS) Forward and DS Backward tests [14]. The CASI consists of 22 items across nine cognitive function domains (“attention, concentration, orientation, short-term memory, long-term memory, language abilities, visual construction, list-generating fluency, abstraction, and judgment”) [15]. The CASI is administered over about 20 min and is scored from 0 to 100 with higher scores indicating higher cognitive function. Prior literature suggests CASI score cutoffs for dementia screening ranged from 71 to 86 depending upon the cultural and educational background of the test-takers [15]. This instrument can be used in people at all levels of cognitive functioning, is designed to be used in different cultural contexts, and was available in both Spanish and Chinese versions [15]. The CASI was selected as the primary outcome test for this analysis because it is a global measure of cognitive function. The Digit Symbol Coding, DS Forward, and DS Backward tests were secondary outcome measures. The Digit Symbol Coding test involves substituting symbols for numbers as quickly as possible over a span of two minutes [16]. This test measures one cognitive function domain, the complex attention domain, via the subdomains of sustained attention and processing speed [17]. The DS Forward Test, in which a person is asked to repeat lists of numbers of increasing length after hearing them, is a measure of complex attention [17, 18]. The DS Backward Test, in which a person repeats a list of numbers backward after hearing them, is a measure of the working memory subdomain of the executive function domain [17, 18]. Other key variables collected at exam 6, which were considered as potential confounders, included family income, alcohol use, and anthropometry measures including body mass index and waist circumference.

A median of 17 months after the exam 6 clinic visit, in 2018–2019, 1062 participants in the MESA Atrial Fibrillation ancillary study returned for an MRI of the brain and upper airway, conducted while the participant was awake [19]. To allow for fat quantitation, the upper airway MRI included a Dixon imaging sequence [20]. All subjects gave informed consent and institutional review boards at all MESA sites approved this study. The MRI images that contained the upper airway were examined by one experienced reader and both soft tissue (31 measurements) and craniofacial measurements (36 measurements) were calculated from each scan slice using an image analysis program [21]. Reproducibility of upper airway measurements using this method was very high, as most intraclass correlations were greater than 0.8 [20].

### Statistical analysis – variable selection

We used lasso (Least Absolute Shrinkage and Selection Operator) regression to select reduced sets of measures most associated with cognitive function from among the 67 upper airway measures and 19 sleep study measures. Lasso regression is a data reduction machine learning technique that shrinks small coefficients to zero and thus removes from the model the measures least associated with the outcome [22]. Thus, lasso regression prevents overfitting and selecting variables that are not associated with the outcome, which can be a drawback of other machine learning methods like ridge regression or decision trees. Lasso performs well even when measures are highly correlated, though if a set of measures are highly correlated, lasso can randomly select one of the set, which can lead to variability of selected measures across imputations. The 19 sleep study measures we considered were selected based on their associations with cognitive function in previous reports [23, 24]. The complete lists of candidate measures are found in Supplemental Table 1. Skewed upper airway and sleep study measures were log base 2 transformed, and these included tongue fat volume and AHI. Participants missing data for a particular cognitive test outcome were excluded from models for that outcome. The median (interquartile range) number of missing upper airway and sleep study variables was 1 (IQR 0–2). Multiple imputation with chained equations (5 imputations) was used to account for missing exposure and covariate data.

We conducted the data reduction steps separately for upper airway measures and sleep study measures. For the upper airway measures, lasso models were used to select measures from the list of 67 candidate upper airway measures without inclusion of sleep study measures. Five-fold cross validation was used to select the lasso regularization parameter with the best model performance as measured by the mean squared error. Using this regularization parameter, the final set of upper airway measures was created by fitting a lasso model on the entire sample. All models were adjusted for the confounders of age, gender, race/ethnicity, study site, current alcohol use, education, income, language spoken, and *APOE* genotype, which are known to be associated with both the exposures and outcomes. Upper airway variables selected in more than half of the imputations were included in further models. The same process was repeated for the 19 sleep study measures without inclusion of the upper airway measures.

Another machine learning technique, Bayesian Model Averaging (BMA), was used as a confirmatory variable selection technique. BMA calculates the probability that each candidate exposure variable will be included in the best fitting model and can work well with correlated variables [25–27]. Like the lasso models, separate BMA analyses were run for the upper airway and sleep variable lists, and the same confounders were included in both BMA models. Variables with a greater than 50% average probability of being in the best fitting model across the five imputations were considered as selected by this technique. Results from the lasso and BMA analyses were considered together in selecting the lists of upper airway and sleep measures for the next step of the analysis [28].

### Statistical analysis – variable association evaluation

The primary model coefficients and p-values were assessed by fitting four linear regression models using all five imputations including the selected upper airway measures, one for each of the four cognitive function tests as the outcome. The linear models were adjusted for the same covariates as the lasso and BMA models. To assess whether the association of upper airway anatomy with cognition is mediated by sleep study measures, another linear regression was run including the selected upper airway measures and the selected sleep study measures plus the standard sleep apnea defining variable, AHI, and the coefficients were compared between the reduced and expanded models. We conducted 2 sensitivity analyses. The first sensitivity analysis added adjustment for body mass index (BMI) and waist circumference to evaluate how much of the impact of upper airway measures remained after accounting for body size, and the second excluded the 2% of participants with a change of > 5 BMI units between the time of the sleep study and the upper airway MRI (between MESA exams 5 and 6). To assess possible effect modification, the primary models were stratified by sex and race/ethnicity and multiplicative interaction terms were used to determine statistical significance, with each racial/ethnic group compared against the other three groups combined.

To assess each step of the hypothesized causal pathway, we also examined the association of upper airway measurements with sleep apnea and the association of sleep measures with cognitive function. In a single multivariable model, the association of each of the selected upper airway measurements with sleep apnea as measured by the AHI was also assessed via linear regression. Using linear regression, the individual associations of the Epworth Sleepiness Scale (ESS), percentage of sleep time at less than 90% oxygen saturation, and AHI were examined in relation to the four cognitive function tests, as previously studied in MESA [24]. These models were adjusted for age, gender, race/ethnicity, study site, current alcohol use, education, income, language spoken, and *APOE* genotype. P-values less than 0.05 were considered significant, and all analyses were conducted in STATA 17 and R 4.1.2.

## Results

From among the 3303 participants who attended exam 6, 591 with data on sleep study measurements, cognitive function, and upper airway anatomy were included in this analysis (Supplemental Fig. 1). The included participants had a similar sex and race/ethnicity composition as the 2712 participants not included, but were on average slightly younger, and had higher family income and higher education level (Supplemental Table 2).

Of the 591 participants included in the analysis, 54% were female, the mean age at exam 6 was 72 years, and 26% of participants were Black, 15% Chinese-American, 19% Hispanic, and 40% White (Table 1). A majority of participants (79%) had an AHI of 5 or more, and 41% had an AHI of 15 or more. The average and standard deviation of selected upper airway measure averages and the cognitive function test scores are displayed in Table 1.

**Table 1:** Characteristics of included participants: demographics, confounders, exposures, and outcomes

Variable	Mean (SD) or N, (%)	N (%) Missing
N	591	
Age	72 (8)	0 (0%)
Race/ethnicity		0 (0%)
Black	155 (26%)	
Chinese-American	91 (15%)	
Hispanic	111 (19%)	
White	234 (40%)	
Gender		0 (0%)
Female	322 (54%)	
Male	269 (46%)	
Language spoken		0 (0%)
English	480 (81%)	
Spanish	47 (8%)	
Chinese	64 (11%)	
Family Income >$50,000 per year	314 (55%)	22 (4%)
Education, College or more	265 (45%)	2 (< 1%)
Current alcohol use	270 (46%)	0 (0%)
*APOE4* carrier	152 (27%)	25 (4%)
BMI (kg/m^2^)	28 (5)	0 (0%)
Waist circumference (cm)	98 (13)	0 (0%)
Soft palate volume (mL)	10.6 (2.7)	0 (0%)
Upper facial height (mm)	47 (4)	0 (0%)
Maxillary divergence (degrees)	56 (5)	0 (0%)
Percentage of tongue fat	20 (3)	55 (9%)
Apnea hypopnea index plus arousals (events per hour)	17 (15)	13 (2%)
Percentage of sleep time < 90% oxygen saturation	2 (5)	0 (0%)
Wake after sleep onset time (minutes)	83 (56)	0 (0%)
Epworth sleepiness scale	6 (4)	10 (2%)
CASI score	91 (7)	20 (3%)
Digit Span Forward score	10 (3)	3 (1%)
Digit Span Backward score	6 (2)	3 (1%)
Digit Symbol Coding score	53 (18)	47 (8%)

CASI = Cognitive Abilities Screening Instrument

### Association of upper airway variables with cognitive test scores

Specific upper airway anatomy measures were selected via lasso and BMA models as potentially having strong associations with CASI and DS Forward scores, but none were selected for the DS Backward or Digit Symbol Coding scores. For the CASI outcome, lasso models consistently selected soft palate volume, maxillary divergence (the angle formed by the incisors and the maxillary tuberosities), upper facial height (the distance from the top of the nose to the bottom of the nose) (Supplemental Figs. 2–4), and four other measures (mandible width at canine, retropalatal airway volume, retropalatal intermandibular volume, and retroglossal airway minimum anterior-posterior distance). The BMA models selected soft palate volume and maxillary divergence as most strongly associated with the outcome (Table 2). Upper facial height was close to being selected by the BMA models with probability of being in the best model of 43%. Accounting for the overlap in selected variables, soft palate volume, maxillary divergence, and upper facial height were considered the key measures going forward. No sleep study measures were selected by either the lasso or BMA models for the CASI.

**Table 2:** Selected upper airway and sleep variables in lasso and Bayesian Model Averaging models for associations with scores on four cognitive function tests

	Cognitive Function Tests			
	CASI	DS Forward	DS Backward	Digit Symbol Coding
Upper airway measures selected in 3 + imputations – lasso models	Soft palate volume Upper facial height Maxillary divergence Mandible width at canine Retropalatal intermandibular volume Retroglossal airway minimum anterior-posterior distance Retropalatal airway volume	Tongue fat percentage Retropalatal airway volume Genioglossus tongue volume Retroglossal lateral wall volume Distance from hyoid to C3 Palatal plane – horizontal angle Maxillary divergence Nasopharyngeal box area Retroglossal intermandibular volume including airway	None	None
Upper airway measures with > 50% probability of inclusion in best fitting BMA model	Soft palate volume Maxillary divergence	Tongue fat percentage	None	Retroglossal airway volume
Sleep study measures selected in 3 + imputations – lasso models	None	None	None	None
Sleep study measures with > 50% probability of inclusion in best fitting BMA model	None	None	None	None

*All models additionally adjusted for age, gender, race/ethnicity, site, current alcohol use, education, income, language spoken, and *APOE4* carrier status

CASI = Cognitive Abilities Screening Instrument, DS = Digit Span

To obtain estimates of association coefficients and test for statistical significance for the machine learning selected variables, a linear regression model was used and found that larger soft palate volume, longer upper facial height, and greater maxillary divergence were significantly associated with higher CASI score indicating better cognition (Table 3). The coefficients were small, with a 1 standard deviation (SD) increase in these variables being associated with a 0.83, 0.75, and 0.70 point higher CASI score, respectively. The four upper airway variables selected by the lasso model but not by the BMA model were not significant in a linear regression analysis (Supplemental Table 3). Adjusting for AHI very slightly attenuated the association of soft palate volume with CASI score, from 0.83 to 0.76 (Table 3). Similarly, adjusting for BMI and waist circumference, and excluding participants with a > 5 BMI change (N = 11 excluded) from exam 5 to exam 6 very minimally attenuated these associations (Table 3).

**Table 3:** Multiple linear regression models for the association of the three upper airway variables selected by lasso and Bayesian Model Averaging models with CASI score (primary analysis), the primary analysis plus AHI, and two sensitivity analyses

	Coefficient*	95% CI		P-value
Primary analysis				
Soft palate volume (per sd)	0.83	0.25	1.41	0.005
Upper facial height (per sd)	0.75	0.17	1.33	0.01
Maxillary divergence (per sd)	0.70	0.12	1.29	0.02
Primary analysis plus AHI				
Soft palate volume (per sd)	0.76	0.18	1.34	0.01
Upper facial height (per sd)	0.74	0.17	1.32	0.01
Maxillary divergence (per sd)	0.74	0.15	1.33	0.01
AHI (events per hour, per doubling)	0.40	-0.02	0.82	0.06
Sensitivity analysis 1 (including adiposity)				
Soft palate volume (per sd)	0.79	0.20	1.37	0.009
Upper facial height (per sd)	0.73	0.15	1.31	0.01
Maxillary divergence (per sd)	0.71	0.12	1.30	0.02
BMI (per sd)	0.95	-0.15	2.05	0.09
Waist circumference (per sd)	-0.75	-1.81	0.31	0.2
Sensitivity analysis 2 (excluding 11 participants with > 5 BMI change between exams 5 and 6)				
Soft palate volume (per sd)	0.79	0.21	1.36	0.007
Upper facial height (per sd)	0.76	0.18	1.34	0.01
Maxillary divergence (per sd)	0.64	0.05	1.22	0.03

*All models additionally adjusted for age, gender, race/ethnicity, site, current alcohol use, education, income, language spoken, and *APOE4* carrier status

CASI = Cognitive Abilities Screening Instrument

In stratified linear regression analyses, different selected upper airway measurements were significantly associated with higher CASI score in the four racial/ethnic groups. In Black participants, soft palate volume was the only significantly associated upper airway variable and had a coefficient of 1.60 higher CASI score per SD higher soft palate volume. In Chinese-American participants, maxillary divergence was the only significantly associated upper airway variable and had a coefficient of 1.67 higher CASI score per SD higher maxillary divergence. In White participants upper facial height was the only significantly associated upper airway variable and had a coefficient of 1.43 higher CASI score per SD higher upper facial height (Table 4). None of the three upper airway variables were significantly associated with CASI score in the Hispanic participants. However, none of these differences by race/ethnicity were significant in tests for interaction (p > 0.1).

**Table 4:** In subgroups defined by race/ethnicity and sex, association of selected upper airway measures with CASI score using a single multiple linear regression model for each subgroup

	Coefficient*	95% CI		P-value
Black participants (N = 147)				
Soft palate volume (per sd)	1.60	0.48	2.72	0.005
Upper facial height (per sd)	0.39	-0.82	1.60	0.5
Maxillary divergence (per sd)	0.72	-0.49	1.93	0.2
Chinese-American participants (N = 88)				
Soft palate volume (per sd)	0.68	-0.69	2.04	0.3
Upper facial height (per sd)	0.10	-1.42	1.63	0.9
Maxillary divergence (per sd)	1.67	0.17	3.18	0.03
Hispanic participants (N = 108)				
Soft palate volume (per sd)	0.62	-1.24	2.48	0.5
Upper facial height (per sd)	0.11	-1.61	1.84	0.9
Maxillary divergence (per sd)	0.89	-0.66	2.44	0.3
White participants (N = 228)				
Soft palate volume (per sd)	0.07	-0.76	0.89	0.9
Upper facial height (per sd)	1.43	0.63	2.23	0.001
Maxillary divergence (per sd)	0.41	-0.46	1.28	0.4
Female participants (N = 312)				
Soft palate volume (per sd)	1.78	0.77	2.78	0.001
Upper facial height (per sd)	0.54	-0.37	1.45	0.2
Maxillary divergence (per sd)	1.13	0.24	2.03	0.01
Male participants (N = 259)				
Soft palate volume (per sd)	0.18	-0.49	0.85	0.6
Upper facial height (per sd)	0.83	0.10	1.56	0.03
Maxillary divergence (per sd)	0.39	-0.38	1.16	0.3

*All models additionally adjusted for age, gender, site, current alcohol use, education, income, language spoken, and *ApoE4* carrier status

CASI = Cognitive Abilities Screening Instrument

In models stratified by sex, differences in the measures significantly associated with CASI were also seen. Soft palate volume (coefficient 1.78 per SD) and maxillary divergence (coefficient 1.13 per SD) were significantly positively associated with CASI score in the female participants, while the significant variable in the male participants was upper facial height (coefficient 0.83 per SD). Tests for interaction of these upper airway measures by sex were significant for soft palate volume (p = 0.006) but not for maxillary divergence or upper facial height (p > 0.5).

For the DS Forward outcome, tongue fat percentage and eight other upper airway measures were selected in 3 of 5 imputations via lasso models. Only tongue fat percentage was selected in BMA models, and accounting for the overlap in selected variables, only tongue fat percentage was considered the key measure going forward. Tongue fat percentage was not significantly associated with DS Forward score in linear models, as the difference in DS Forward score per doubling of tongue fat percentage was 1.04 (95% confidence interval − 0.10, 2.17). No upper airway measures were selected by the lasso or BMA models for the DS Backward or Digit Symbol Coding tests, and no sleep study measures were selected for any of the three secondary cognitive function outcomes (Table 2).

### Association of upper airway variables with sleep apnea

Larger soft palate volume was significantly associated with higher AHI, while upper facial height and maxillary divergence were not significantly associated with AHI (Table 5). None of the selected upper airway variables were significantly associated with the Epworth Sleepiness Scale or the percentage of sleep time at less than 90% oxygen saturation.

**Table 5:** Single multiple linear regression model for the association of the three upper airway variables selected by lasso and Bayesian Model Averaging models with log2 transformed apnea-hypopnea index

	Coefficient*	95% CI		P-value
Soft palate volume (per sd)	0.14	0.02	0.25	0.02
Upper facial height (per sd)	0.01	-0.10	0.13	0.8
Maxillary divergence (per sd)	-0.09	-0.20	0.03	0.1

*Model additionally adjusted for age, gender, race/ethnicity, site, current alcohol use, education, income, language spoken, and *APOE4* carrier status

### Association of sleep study variables with cognitive test scores

In linear regression models, higher AHI was marginally associated with higher CASI score indicating better cognitive function (coefficient per AHI doubling 0.43 [0.01, 0.85], p = 0.04) while AHI, the Epworth Sleepiness Scale, and the percentage of sleep time with less than 90% oxygen saturation were not significantly associated with any of the other cognitive function tests.

## Discussion

This exploratory analysis found that three upper airway anatomy measures were significantly but weakly associated with global cognitive function as measured by the CASI score: soft palate volume, maxillary divergence, and upper facial height. However, these three upper airway measures were not associated with the DS Forward, DS Backward, or Digit Symbol Coding test scores. In addition, the association with CASI score of one of the measures, soft palate volume, was in the opposite direction to our hypothesis. Also contrary to hypothesis, adjusting for sleep apnea severity as measured by the AHI did not attenuate the upper airway associations with cognitive test performance, and no measures of sleep, including measures of duration, fragmentation, or hypoxemia, were selected as significantly associated with cognitive function. Thus, the results do not support that the upper airway and cognitive function associations are working through the mechanism of sleep apnea-based hypoxemic damage to the brain, though it is possible that the key sleep measure mediator was not measured in this study. It is unclear through which biological pathways soft palate volume, maxillary divergence, and upper facial height are associated with cognitive function. Adjusting for body size via BMI and waist circumference did not attenuate the strength of these associations. Where fat is stored in the body has been shown to be associated with cognitive function [29]. It is possible that this study did not collect the most relevant fat measure or that higher soft palate volume may better capture relevant fat deposition than measures such as BMI or waist circumference. It is also possible that the identified upper airway measures may be markers for other unmeasured health conditions. These results do not suggest that clinical intervention on upper airway anatomy characteristics would provide benefits for cognitive functioning. The overlap in variables selected by lasso and BMA models, as well as previous studies reporting that the selected variables are associated with sleep apnea, provides reinforcement that the selected variables are plausible links between upper airway anatomy and cognitive function. These associations require additional investigation, as there are no previous studies of the association of upper airway measures with cognitive function test scores.

Regarding the association of upper airway dimensions with sleep apnea, our finding that larger soft palate volume was significantly associated with higher AHI matches what was found in previous studies. In two meta-analyses of the associations between lateral X-ray data and obstructive sleep apnea, both found that larger soft palate area, and larger tongue area were associated with obstructive sleep apnea [5, 6]. Additionally, a case-control study reported that larger soft palate length and larger hyoid-mandibular distance were associated with sleep apnea even after matching on BMI [30], and another study of obese persons reported that those with a higher AHI also had larger soft palate volumes and tongue volumes [31]. Two previous studies focused on tongue fat as an important upper airway anatomy parameter. The first found that compared to age, sex, race, and BMI matched controls, persons with sleep apnea had significantly larger tongue fat volumes [32]. In the second study, which examined weight change in persons with sleep apnea, tongue fat volume was longitudinally associated with AHI [20]. Based on these studies, we had hypothesized that lower tongue fat would be associated with better cognition, but the present analysis did not find tongue fat to be significantly associated with any of the cognitive function tests.

Regarding the association of sleep study measures with cognitive function, our results add to the mixed results in the existing literature. Contrary to our hypothesis, our analysis found that higher AHI was weakly associated with better CASI score. Two studies found no association between AHI and several measures of cognitive function [10, 33]. Nikodemova et al. found that higher severity of sleep disordered breathing was associated with poorer cognitive function in persons with an Alzheimer’s risk gene [3]. In 1752 MESA participants, Johnson et al. found that self-reported sleepiness and a higher percentage of sleep time with low blood oxygen, but not elevated AHI, were associated with lower DS Forward test scores, but not with CASI score [24]. Compared with the Johnson study, our analysis had a smaller sample size, higher average CASI score and lower median AHI. Additionally, the studies by Nikodemova and Johnson found the largest associations in *APOE4* variant carriers, but our analysis did not focus on variant carriers and included too few *APOE4* carriers to have sufficient power to find significant associations in this subgroup.

Several studies have reported differences by race in the association of upper airway anatomy with sleep apnea [21, 34, 35]. In this analysis, we examined differences by race/ethnicity in the association of upper airway anatomy with cognitive function. The results of this analysis suggested possible differences by race/ethnicity but these differences did not reach statistical significance, possibly due to lack of power. Differences by sex in the associations of upper airway anatomy with cognitive function were also found in this study, as larger soft palate volume was significantly associated with higher CASI score in women but not men. This difference by sex requires additional study. As with the overall results, these results do not support employing upper airway anatomy clinical interventions in subgroups of race/ethnicity or sex to improve cognitive functioning.

This exploratory analysis has a number of strengths, including a large sample size from a racially diverse cohort study, accurate and reproducible measurement of upper airway anatomy by MRI, and analyses utilizing two different machine learning techniques to perform variable selection. Limitations include lack of power within racial/ethnic groups, potential unmeasured residual confounding, potential selection bias of participants with higher cognitive function test scores and lower AHI, that sleep study measurements were taken about six years before the cognitive function measures, and that upper airway anatomy was measured about 17 months after cognitive function. While all study participants were untreated for sleep apnea at the time of the sleep study, sleep apnea treatment at the time of the cognitive function tests was not measured. Additionally, the assessed cognitive function tests are limited and do not measure all possible cognitive function domains.

## Conclusions

Upper airway anatomy as measured by soft palate volume, maxillary divergence, and upper facial height was significantly associated with global cognitive test performance. The associations with CASI score were small in magnitude: each standard deviation difference in upper airway measures was associated with less than a point of difference in CASI score. By contrast, in this dataset, for each 10 years older age, CASI score was 2.2 points lower. Sleep study measurements did not appear to be the pathway through which these associations were acting. This study does not suggest that clinical intervention on upper airway anatomy characteristics would provide benefits for cognitive functioning. As these analyses were exploratory, further research focusing on the selected upper airway measures should be pursued.

### Electronic supplementary material

Below is the link to the electronic supplementary material.

Supplementary Material 1

## Data Availability

The data that support the findings of this study are available from the MESA Coordinating Center but restrictions apply to the availability of these data, which were used under license for the current study, and so are not publicly available. MESA data are however available from the MESA data coordinating center upon reasonable request and approval.

## Abbreviations

AHI: Apnea hypopnea index
CASI: Cognitive Abilities Screening Instrument
SD: Standard deviation
MRI: Magnetic resonance imaging
MESA: Multi-Ethnic Study of Atherosclerosis
DS: Digit span
Lasso: Least Absolute Shrinkage and Selection Operator
IQR: Interquartile range
BMA: Bayesian Model Averaging
BMI: Body mass index
ESS: Epworth Sleepiness Scale
