# Supplementary material for: The association of upper airway anatomy with cognitive test performance: the Multi-Ethnic Study of Atherosclerosis
Source: BMC Neurol. 2023 Oct 31;23:394. doi: 10.1186/s12883-023-03443-9 (PMC10617161; doi:10.1186/s12883-023-03443-9)
Supplement: Supplementary file 1 — Supplementary Material 1 [file 12883_2023_3443_MOESM1_ESM.pdf]

Supplemental Table 1. List of upper airway and sleep study measurements examined in this analysis(21)

Upper Airway Anatomy - Soft tissue from upper airway MRI
Mandible volume
Retropalatal (RP) airway volume
Retroglossal (RG) airway volume
Epiglottis volume
Soft palate volume
Tongue volume
Pterygoid volume
Fat pad volume
RP lateral wall volume
RG lateral wall volume
RP intermandibular volume
RG intermandibular volume
RP intermandibular volume extended to spine
RG intermandibular volume extended to spine
Total intermandibular volume
Total intermandibular volume extended to spine
Minimum cross-sectional area in the RP and RG regions
Minimum anterior-posterior and lateral dimensions in RP and RG regions
Total airway volume
Airway cross-sectional area
Length of airway
RP airway cross-sectional area
RG airway cross-sectional area
Tongue fat volume
Soft palate fat volume
Tongue fat percentage
Soft palate fat percentage
Upper Airway Anatomy - Craniofacial from upper airway MRI
SNA angle
SNB angle
ANB angle
Saddle angle
Anterior cranial base vs horizontal angle
Palatal plane vs horizontal angle
Sella-hyoid distance
Retropogonion-C3 distance
Retropogonion-hyoid distance
Hyoid-C3 distance
PNS-Anterior arch atlas distance
Upper facial height
Lower facial height
Anterior facial height
Facial height ratio
Mandibular divergence

Mandible width at canine<br>Mandible width at first premolar<br>Mandible width at second premolar<br>Mandible width at first molar<br>Mandible width at second molar<br>Mandible width at inner gonion<br>Mandible width at condyle<br>Mandible depth<br>Maxillary divergence<br>Maxilla width at canine<br>Maxilla width at first premolar<br>Maxilla width at second premolar<br>Maxilla width at first molar<br>Maxilla width at second molar<br>Maxilla width at tuberosity<br>Maxilla depth<br>Mandible inner surface area<br>Nasopharyngeal box area<br>Oropharyngeal box area<br>Naso-oropharyngeal box area
Sleep Architecture<br>REM (Rapid Eye Movement) minutes<br>Sleep efficiency<br>Sleep maintenance efficiency<br>Total sleep time<br>Wake after sleep onset time<br>% time in N1<br>% time in N2<br>% time in N3<br>Arousal index
Sleep Hypoxemia<br>Hypoxic burden (total oxygen desaturation percent-minutes per hour of sleep)<br>Oxygen nadir non-REM<br>Oxygen nadir REM<br>% sleep time <90% oxygen saturation<br>Apnea-hypopnea index (4%)<br>Apnea-hypopnea index (4%) plus arousals
Sleepiness from Self-Report Questionnaire<br>Sleep duration (weekday)<br>Epworth sleepiness scale<br>Women’s Health Initiative Insomnia Rating Scale (WHIIRS)<br>Snoring frequency

Supplemental Table 2. Characteristics at exam 6 of MESA participants included and not included in this analysis

	Exam 6 MESA participants not included in analysis	Exam 6 MESA participants included in analysis
N	2712	591
Age in years (Mean, SD)	75 (9)	72 (8)
Race/ethnicity		
Black	696 (26%)	155 (26%)
Chinese-American	329 (12%)	91 (15%)
Hispanic	599 (22%)	111 (19%)
White	1088 (40%)	234 (40%)
Gender		
Female	1438 (53%)	322 (54%)
Male	1274 (47%)	269 (46%)
Language spoken		
English	2151 (79%)	480 (81%)
Spanish	302 (11%)	47 (8%)
Chinese	259 (10%)	64 (11%)
Family Income >$50,000 per year	1221 (47%)	314 (55%)
Education, College or more	1114 (41%)	265 (45%)

Supplemental Table 3. Association of all lasso-selected upper airway measures with CASI outcome adjusted for race/ethnicity, gender, site, age, language, current alcohol use, income, education, and *APOE4* carrier status

	Coefficient*	95% CI		P-value
Soft palate volume (per sd)	0.69	0.07	1.31	0.029
Upper facial height (per sd)	0.70	0.12	1.28	0.018
Maxillary divergence (per sd)	0.75	0.16	1.33	0.012
Retropalatal airway volume (per doubling)	0.69	-0.11	1.50	0.091
Retropalatal intermandibular volume (per sd)	0.33	-0.30	0.95	0.301
Mandible width at canine (per sd)	-0.49	-1.03	0.05	0.076
Retroglossal airway minimum anterior-posterior distance (per sd)	-0.52	-1.07	0.02	0.061

*All models additionally adjusted for age, gender, race/ethnicity, site, current alcohol use, education, income, language spoken, and *APOE4* carrier status

CASI= Cognitive Abilities Screening Instrument

Supplemental Figure 1. Flowchart of included participants.

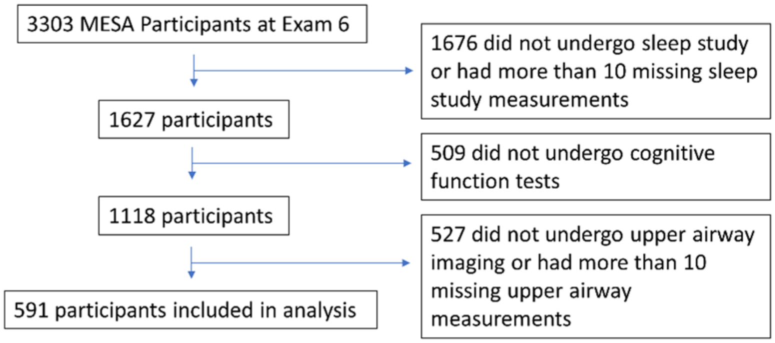

Supplemental figure 2. Soft palate volume.

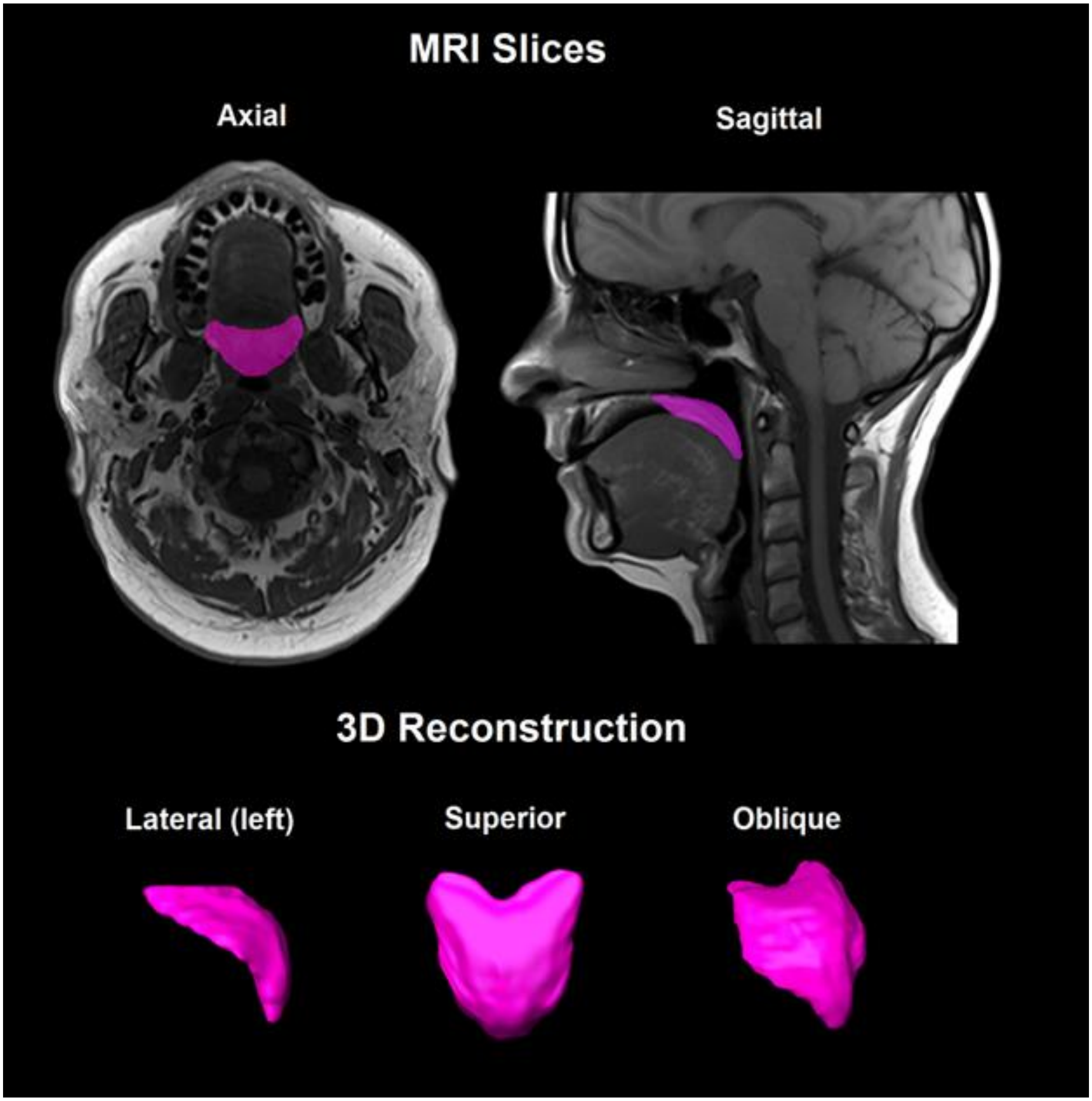

Magnetic resonance images (Axial, Sagittal, T1 Spin Echo) and three-dimensional reconstructions highlighting the volumetric segmentation of soft palate (magenta). The soft palate extends from its origin at the hard palate to its end at the uvula. The presence of the soft palate defines the retro-palatal region of the airway. Volumetric segmentation is performed slice-by slice on axial MRI (3mm slice thickness) using Amira software (Thermofisher Scientific). 3D reconstructions are rendered from segmentation data.

Supplemental figure 3. Upper facial height.

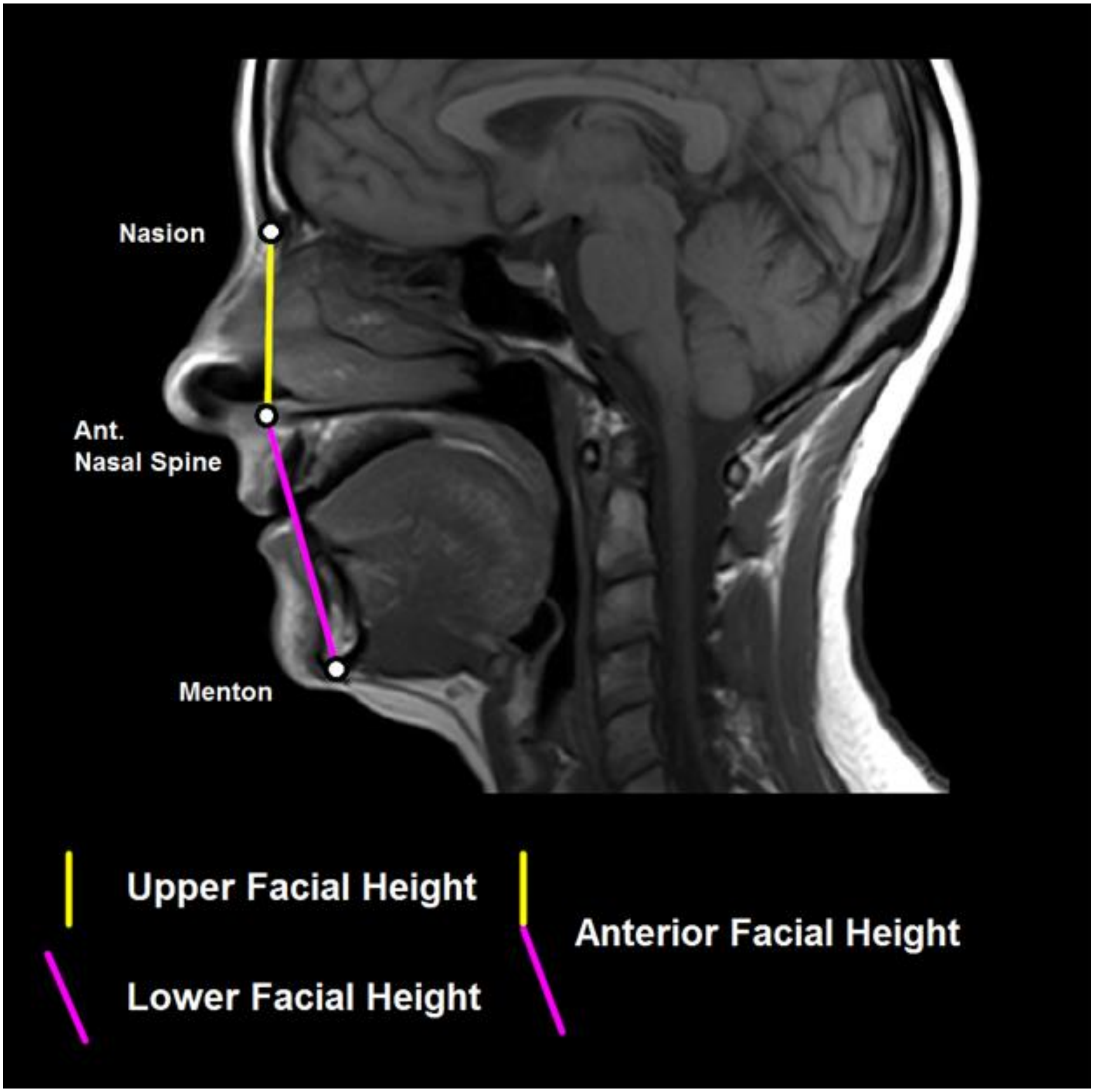

Mid-sagittal magnetic resonance image (T1 Spin Echo) showing facial height measurements. Landmarks are the Nasion, the most anterior aspect of the frontonasal suture; the Anterior Nasal Spine (ANS), the most anterior point of said structure; and the Menton, the most inferior point of the mandibular symphysis. Upper Facial Height measures the distance from Nasion-ANS. Lower Facial Height measures the distance from ANS-Menton. Anterior Facial Height represents the sum of these measures, with which the individual measures are compared to produce ratios.

Supplemental figure 4. Maxillary divergence.

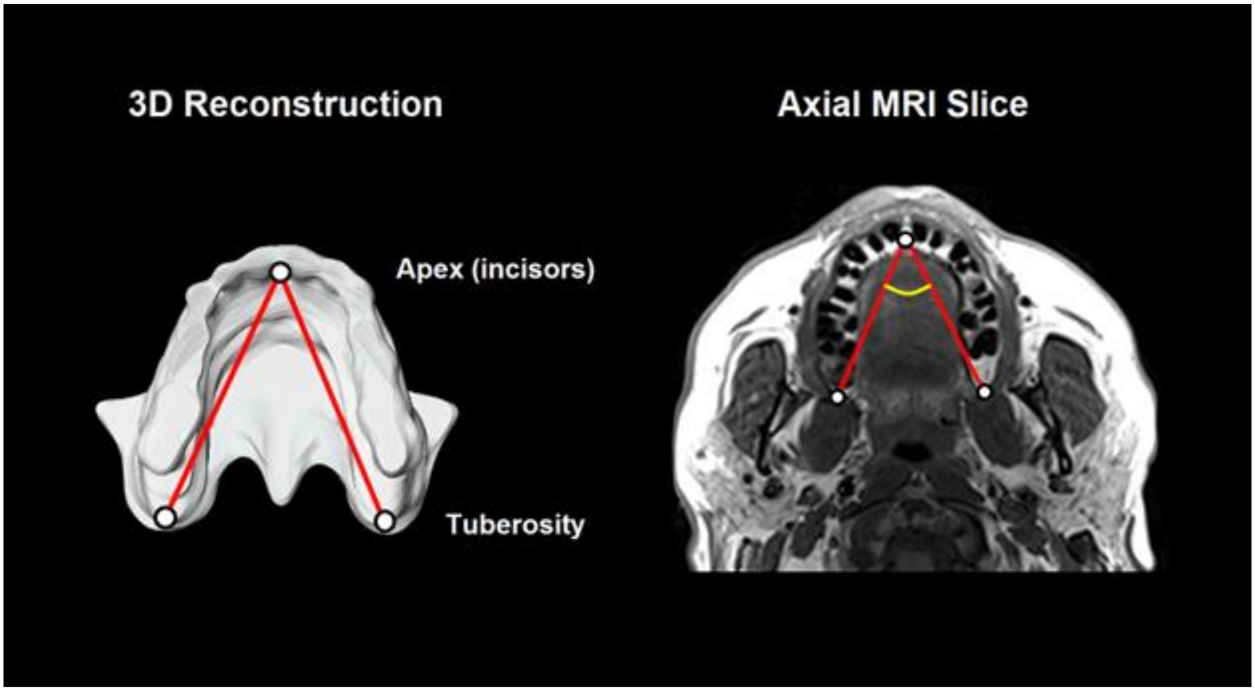

Magnetic resonance image (Axial, T1 Spin Echo) and three-dimensional reconstruction showing the maxillary divergence measurement, the angle characterizing the relative width of the maxilla. Landmarks are the Apex, the midpoint of the central maxillary incisors at the alveolar ridge; and Tuberosities, the most posterior bilateral points of the body of the maxilla. The angle is directly measured on to-scale 3D reconstructions derived from slice-by-slice volumetric segmentation of axial T1 Spin Echo MRI using Amira software (Thermofisher Scientific).
